# Risk scores of incident mild cognitive impairment in a Beijing community-based older cohort

**DOI:** 10.3389/fnagi.2022.976126

**Published:** 2022-10-03

**Authors:** Xin Li, Jianan Xia, Yumeng Li, Kai Xu, Kewei Chen, Junying Zhang, He Li, Zhanjun Zhang

**Affiliations:** ^1^State Key Laboratory of Cognitive Neuroscience and Learning, Beijing Normal University, Beijing, China; ^2^BABRI Centre, Beijing Normal University, Beijing, China; ^3^School of Computer and Information Technology, Beijing Jiaotong University, Beijing, China; ^4^Banner Alzheimer’s Institute, Phoenix, AZ, United States; ^5^Institute of Basic Research in Clinical Medicine, China Academy of Traditional Chinese Medicine, Beijing, China

**Keywords:** mild cognitive impairment, lifestyle-related disease, risk score, cognition, prevention

## Abstract

**Objective:** It is very important to identify individuals who are at greatest risk for mild cognitive impairment (MCI) to potentially mitigate or minimize risk factors early in its course. We created a practical MCI risk scoring system and provided individualized estimates of MCI risk.

**Methods:** Using data from 9,000 older adults recruited for the Beijing Ageing Brain Rejuvenation Initiative, we investigated the association of the baseline demographic, medical history, lifestyle and cognitive data with MCI status based on logistic modeling and established risk score (RS) models 1 and 2 for MCI. We evaluated model performance by computing the area under the receiver operating characteristic (ROC) curve (AUC). Finally, RS model 3 was further confirmed and improved based on longitudinal outcome data from the progression of MCI in a sub-cohort who had an average 3-year follow-up.

**Results:** A total of 1,174 subjects (19.8%) were diagnosed with MCI at baseline, and 72 (7.8%) of 849 developed MCI in the follow-up. The AUC values of RS models 1 and 2 were between 0.64 and 0.70 based on baseline age, education, cerebrovascular disease, intelligence and physical activities. Adding baseline memory and language performance, the AUC of RS model 3 more accurately predicted MCI conversion (AUC = 0.785).

**Conclusion:** A combination of risk factors is predictive of the likelihood of MCI. Identifying the RSs may be useful to clinicians as they evaluate their patients and to researchers as they design trials to study possible early non-pharmaceutical interventions to reduce the risk of MCI and dementia.

## Introduction

Societies comprise an increasing proportion of older adult who, because of age alone, are at an increasing risk of Alzheimer’s disease (AD; Prince et al., 2013). Governments are concerned about the increase in the number of people with AD. Mild cognitive impairment (MCI) is often regarded as an intermediate state from normal cognition to AD. Annual conversion rates from MCI ( >65 years old) to dementia vary from 4% to 31% (Bruscoli and Lovestone, 2004), compared to the prevalence of 5.6% for the older adult. With increasing emphasis on the need to treat incipient dementia at an early stage, it will be important to know which individuals have a high likelihood of a prognosis of MCI since it is a target stage for biomarkers for the early diagnosis and prediction of AD.

Based on a number of large-scale cohort studies from various research groups, there are several factors associated with the risk of MCI. They include education, sex (Mielke et al., 2012), diabetes (Roberts et al., 2010; Geda et al., 2011), depressive symptoms (Gallagher et al., 2018), cardiovascular disease (Roberts et al., 2010), cerebral vascular disease (Knopman et al., 2009), cognition and physical activity and so on. Analyses of the data in Beijing community-based older cohort, confirmed these findings (Li et al., 2013). Note that some identified risk factors for MCI are modifiable, and therefore, potential non-pharmaceutical intervention strategies can be developed to mitigate and minimize these risk factors for MCI.

The establishment of a risk profile for MCI is key and expected upon evidence that the modification of several potent risk factors will reduce the probability of developing MCI and AD. A similar approach has been followed successfully regarding cardiovascular events, diabetes, and mortality (D’Agostino et al., 1994). Risk scores have generally included only a few known factors that are easily measurable. The main use of risk scores is for targeting individuals who are at high risk of the disease (Hall et al., 2003) to change their behavior (modifiable factors) on an individual basis or, more formally, in the context of prevention trials to evaluate the efficacy of these changes. Another practical benefit is that they can be used to distribute easily understandable information about risk factors to the general population. However, its application to dementia has been more limited. In 2006, the Cardiovascular Risk Factors, Aging, and Incidence of Dementia (CAIDE) study developed a score index to predict the risk of dementia on the basis of risk factor profiles present in middle age (Kivipelto et al., 2006). Using 8 years of follow-up data from a New York-based sample, a scoring system was developed for predicting late-onset Alzheimer’s disease risk in older individuals using more commonly available measures. Recently, several risk scores for dementia have been calculated to identify and monitor risk status by targeting modifiable, lifestyle-related risk factors (Schiepers et al., 2018). Li and colleagues developed risk score systems to estimate 5-, 10-, and 20-year dementia risk predictions. This risk score system provides a practical tool because all included predictors are easy to assess by practitioners (Li et al., 2018). Furthermore, dementia risk scores might be useful surrogate outcomes for dementia prevention trials (Coley et al., 2020). To date, however, there has been no study on risk scores for MCI.

The aim of this study was to establish such a risk score system based on data from the BABRI study, and longitudinal outcome data regarding the progression of MCI were used to improve the system. The risk score system could also be used to accurately stratify older adults into different risks of MCI. Defining the role of risk factors will help implement future primary and secondary prevention trials to reduce the incidence of MCI and AD in older adults.

## Methods

### Participants

The Beijing Ageing Brain Rejuvenation Initiative (BABRI) has been previously described (Yang et al., 2021). The institutional review board of Beijing Normal University approved the study. Briefly, the study enrolled Chinese-speaking community-residing subjects aged between 50 and 80 years. Exclusion criteria included severe visual or hearing loss, neurological, psychiatric, or systemic illness, and psychoactive medication use. Individuals who completed the neuropsychological tests but refused to answer the personal information questionnaire for privacy or other reasons were excluded from the analysis of associated factors. Subjects were screened to rule out the presence of dementia. Written informed consent was obtained from each subject at enrolment.

The study recruited more than 9,000 subjects between 2008 and 2018, and the recruitment strategies have been described in our previous studies (Yang et al., 2021). At baseline and subsequent visits, participants were evaluated for demographic, lifestyle, and neuropsychological measures and were classified as NC, MCI, or dementia. Enrolled study participants were followed-up every 2 or 3 years, and as many new participants as possible were continuously recruited. In this research, we used data from 5,921 subjects (NCs and those with MCI) with the complete information needed for the design of this study. Among these 5,921 individuals, there were 921 who were free from MCI at baseline and had two or more follow-up visits. Their data were analyzed to explore the predictive factors related to the conversion to MCI.

### Neuropsychological tests

A group of students well trained by professional neuropsychologists performed neuropsychological testing and collection of personal information questionnaires. The Chinese translation of the Mini-Mental State Examination (MMSE) was used to assess scores that served as an exclusion criterion (Folstein et al., 1975); subjects scoring ≤23 were considered possible dementia patients and were excluded. The subsequent neuropsychological battery tested five cognition domains: (1) episodic memory, tested by the Auditory Verbal Learning test and the Rey-Osterrieth Complex Figure test (ROCF; recall); (2) attention, tested by the Trail Making Test A and the Symbol Digit Modalities Test (SDMT); (3) visuo-spatial ability, tested by the ROCF test (copy) and the Clock-Drawing Test, (4) language, tested by the Category Verbal Fluency Test and the Boston Naming Test; and (5) executive function, tested by the Trail Making Test B and the Stroop Test.

### MCI diagnostic criteria

Consensus diagnoses were assigned by two multidisciplinary experts. The subjects were diagnosed with MCI if they had the following symptoms according to Petersen’s criteria (Petersen and Morris, 2005): (1) subjective memory complaints; (2) preserved normal cognitive functions, as assessed by scores on the MMSE (higher than 23); (3) essentially intact activities of daily living (ADLs) and instrumental activities of daily living (IADLs); (4) not demented; (5) objective abnormal memory impairment based on a cut-off of 1.5 standard deviations (SD) after normative corrections for age and years of formal education; and (6) no objective impairment in other cognitive domains (attention, visuospatial, language and executive function).

### Lifestyle

Leisure activities were defined as activities in which individuals participated for enjoyment that was independent of work and included intellectual activities, physical activities, and social contact activities. Response choices ranged from “never did, or used to, but not in the past year” (score = 1) to “every day” (score = 5). Intellectual activities included reading, writing, taking courses in a senior citizen university, playing chess and card activities, hand crafting, doing calligraphy or taking photos, using a computer or doing crossword puzzles. Physical activities included aerobic exercise, muscular endurance sports, dancing, traditional Chinese martial arts, climbing mountains, skiing, picking fruit, fishing and gardening. Social activities included playing team games, visiting relatives and friends and attending a party. The scores of intellectual activities, physical activities, and social contact activities were then calculated based on their responses to these questions about their leisure activities.

Eating habits were measured based on the Eating Habits Inventory (EHI; Xu et al., 2010), which is a checklist of daily diet preference, such as intake of unrefined cereal grains, vegetables, and nuts and high-calorie and fatty foods. Response choices range from “never” (score = 1) to “always” (score = 4). After converting the choices, higher scores indicated a healthier diet. Life regularity was evaluated by the Life Regularity Self-Rating Inventory (IRSI), which primarily focuses on eating and sleeping habits. Response choices range from “never” (score = 1) to “always” (score = 4).

### Medical history

Medical history included questions on a series of chronic diseases, including hypertension, coronary heart disease, diabetes mellitus, cerebrovascular disease, chronic bronchitis or emphysema, osteoarthritis, and intervertebral disk disease.

### Statistical analysis

Differences between participants with and without MCI regarding their midlife characteristics were described using Student’s *t*-test or the *χ*^2^ test. To individually examine the risk factors, the midlife lifestyle (social, physical activities, etc.), midlife status (income, job, BMI, etc.) and disease factors were each separately included in the logistic regression model together with age, sex, and education. Factors that were significant in the first step were then simultaneously included in a single logistic regression model with stepwise regression procedure. Then, we established risk score (RS) model 1 and model 2 (model 1 + MMSE) for MCI. Risk scores were assigned for each factor from model 1 and model 2. All β coefficients were standardized to make the scores approach an integer value so that the lowest coefficient had a value of 1. Since the lowest β value was 0.184 and its multiplication by 5 makes it approximately 1, all β values were multiplied by 5 and rounded to the closest integer. Therefore, the risk score for an individual was obtained by summing the scores of each risk factor and the range of possible scores (RS1: 0–18 for model 1 and RS2: 0–22 for model 2). Finally, RS model 3 was further constructed based on baseline cognition and longitudinal outcome data regarding the progression of MCI from a sub-cohort with follow-up information.

The predictive accuracy of the model was assessed based on discrimination, which refers to the ability of the index to accurately distinguish between NC and MCI individuals and was assessed using the area under the curve (AUC) of the receiver operating characteristic (ROC) curve, also known as the c statistic. The c statistic may range from 0 to 1: a c statistic of 0.5 indicates that predictive accuracy is no better than chance, while a c statistic of 1 indicates perfect discrimination. We also categorized subjects as having low, moderate, or high scores on the final risk index and calculated the occurrence of MCI within each group.

## Results

### Prevalence of MCI

In this sample, 1,174 subjects (19.8%) were diagnosed with MCI. The MCI group was significantly older and had a lower education level than the group with normal cognition (*t*_age_ = 5.61, *p*_age_ < 0.001; *t*_edu_ = −13.05, *p*_edu_ < 0.001). The sex-specific prevalence was also determined (*χ*^2^ = 21.83, *p* < 0.001), with an MCI prevalence of 22.8% in men and 17.9% in women (Table 1).

**Table 1:** Characteristics of the study sample at baseline.

**Characteristic**	**MCI (*N* = 1,174)**		**NC (*N* = 4,747)**		***t*-test/Chi-Square/*F*-test**	***p***
	**Mean**	**SD**	**Mean**	**SD**		
Age (year)	66.04	7.09	64.76	6.74	5.608	*p* < 0.001
Education (year)	9.99	2.94	11.26	2.99	−13.047	*p* < 0.001
Gender (M/F)	539/635		1,825/2,921		21.825	*p* < 0.001
BMI	24.89	4.27	24.84	4.07	0.005	*p* = 0.942
Married	979 (85.0%)		4,070 (88.0%)		7.521	*p* = 0.006
Smoking	720 (68.8%)		3,447 (77.0%)		30.704	*p* < 0.001
Drinking	683 (72.1%)		3,016 (76.7%)		8.659	*p* = 0.003
Jobscore	2.60	1.41	2.89	1.43	6.853	*p* = 0.009
Imcomelevel	6.02	3.07	6.97	3.03	31.355	*p* < 0.001
UCLA	34.87	9.90	34.07	9.12	6.091	*p* = 0.014
GDS	8.70	6.38	7.29	5.75	40.777	*p* < 0.001
Intellectual activities	1.70	0.56	2.06	0.64	156.310	*p* < 0.001
Physical activities	2.40	0.51	2.57	0.52	55.747	*p* < 0.001
Social contact activities	1.79	0.61	1.99	0.67	35.451	*p* < 0.001
Eating Habit	3.23	0.35	3.26	0.36	2.209	*p* = 0.137
Life regularity	3.62	0.39	3.64	0.37	6.492	*p* = 0.011
Diabetes mellitus	267 (23.0%)		975 (20.9%)		2.548	*p* = 0.110
Hypertension	592 (51.0%)		2,230 (47.7%)		4.035	*p* = 0.045
Hyperlipidemia	295 (25.4%)		1,424 (30.5%)		11.421	*P* = 0.001
Cerebrovascular disease	238 (20.5%)		653 (14.0%)		30.677	*p* < 0.001
Coronary heart disease	199 (17.2%)		727 (15.6%)		1.761	*p* = 0.185

### Potential risk factors for MCI

Differences in the medical histories between the group with MCI and the group with normal cognition were examined. Several common geriatric diseases were considered. Using the chi-square (*χ*^2^) test, only vascular risk factors affected MCI. The prevalence of hypertension (*χ*^2^ = 4.04, *p* = 0.045), hyperlipidemia (*χ*^2^ = 11.42, *p* = 0.001), and cerebrovascular disease (*χ*^2^ = 30.68, *p* < 0.001) were each higher in the group with MCI than in the group with normal cognition (Table 1).

There were significant lifestyle differences between the MCI and NC groups. The MCI patients had lower scores in intellectual activities (*t* = 156.31, *p* < 0.001), physical activities (*t* = 55.75, *p* < 0.001), social contact activities (*t* = 35.45, *p* < 0.001) and life regularity (*t* = 6.49, *p* = 0.011). There was no significant difference in eating habits (*t* = 2.21, *p* = 0.137) between the two groups. Compared with the NCs, the MCI patients had lower income levels (*t* = 31.36, *p* < 0.001) and job scores (*t* = 6.85, *p* = 0.009). The MCI patients also showed high depressive (*t* = 40.78, *p* < 0.001) and loneliness (*t* = 6.09, *p* = 0.014) symptoms (Table 1).

### Risk scores of MCI

**Figure 1:**
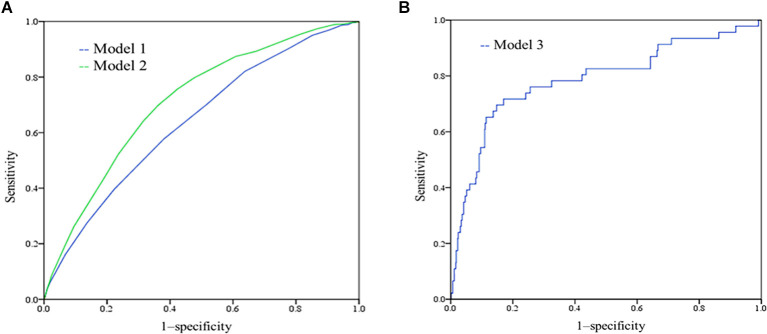
**(A)** Receiver-operating characteristic (ROC) of model 1 and model 2. The AUC for model 1 was 0.640 (95% CI 0.620–0.660), and the AUC for model 2 was 0.712 (0.694–0.730); **(B)** ROC curves showing the performance of the mild cognitive impairment (MCI) risk scores (model 3) in predicting the incident MCI.

A wide range of factors were associated with an increased risk of dementia after adjustment for age, sex and education and were considered for inclusion in the MCI risk index. These included demographic factors (older age, lower education, lower income); cerebrovascular disease; healthy lifestyle (low intellectual and physical activity); and low MMSE performance. Table 2 shows the coefficients of variables in the final logistic regression model. The AUC for risk score model 1 was 0.640, and that for model 2 was 0.712, as shown in Figure 1A. The risk scores in the NC group were significantly lower than those in the MCI group with both models.

**Table 2:** Logistic regression models for MCI risk according to the selected risk factor.

	**Model 1**						**Model 2**					
	**β**	***P***	**OR**	**CI**		**Score**	**β**	***P***	**OR**	**CI**		**Score**
**Age**												
50–60	0 (reference)		1			**0**	0 (reference)		1			**0**
61–65	0.184	0.104	1.202	0.963	1.501	**1**	0.123	0.249	1.131	0.917	1.395	**1**
> 66	0.403	0.000	1.497	1.211	1.851	**2**	0.239	0.019	1.270	1.040	1.552	**1**
**Education**												
> 12 years	0 (reference)		1			**0**						**0**
≤ 12 years	0.381	0.001	1.463	1.176	1.821	**2**	0.316	0.003	1.372	1.112	1.692	**2**
**Gender**												
Female	0 (reference)		1			**0**						**0**
Male	0.446	0.000	1.561	1.323	1.842	**2**	0.353	0.000	1.424	1.217	1.666	**2**
**Cerebrovascular**												
No	0 (reference)		1			**0**						**0**
Yes	0.570	0.000	1.769	1.446	2.164	**3**	0.570	0.000	1.768	1.459	2.142	**3**
**Income level**												
High	0 (reference)		1			**0**	0 (reference)		1			**0**
Low	0.523	0.000	1.687	1.424	1.999	**3**	0.440	0.000	1.552	1.321	1.824	**2**
**Intellectual Activity**												
Active	0 (reference)		1			**0**	0 (reference)		1			**0**
Inactive	0.858	0.001	2.359	1.452	3.834	**4**	0.714	0.002	2.042	1.291	3.229	**4**
**Physical Activity**												
Active	0 (reference)		1			**0**	0 (reference)		1			**0**
Inactive	0.409	0.002	1.506	1.168	1.942	**2**	0.374	0.003	1.454	1.140	1.854	**2**
**MMSE**												
> 27							0 (reference)		1			**0**
≤ 27							1.169	0.000	3.220	2.739	3.786	**6**
**Intercept**	−3.707	0.000					−3.916	0.000				

Different weight points were given for each risk factor based on their standardized β-coefficients in the logistic regression model, and score sheets were developed to predict MCI. The model 1 score (RS1) ranged from 0 to 18, and the model 2 score (RS2) ranged from 0 to 22. Table 3 shows the percentages of MCI at different risk score levels. A total of 32.8% of the population had a value of 11.5 or greater in RS1, while this cut-off had a sensitivity of 0.578 and a specificity of 0.619. The accuracy for risk score model 1 was 64%. A total of 42.8% of the population had a value of 13.5 or greater in RS21, while this cut-off had a sensitivity of 0.697 and a specificity of 0.639. The accuracy for risk score model 2 was 65%.

**Table 3:** The occurrence of MCI according to the categories of the MCI risk scores.

	**Model 1**	**Model 2**
Risk Score = 0	2.98%	1.78%
Risk Score = 12	22.46%	15.34%
Risk Score = 14	29.63%	21.02%
Risk Score = 18	47.08%	36.45%
Risk Score = 22	—	55.28%

### Prevalence of incident MCI and potential risk factors

There were 1,036 participants who had longitudinal follow-up visits. At baseline, 921 participants were classified as normal cognition (NC); among them, 849 participants maintained NC status, and 72 (7.8%) developed MCI. Compared with the NC-NC group, the NC-MCI group was older (*t* = 2.64, *p* = 0.009) and had a higher education level (*t* = −2.04, *p* = 0.042; Table 4). Regarding a healthy lifestyle, the NC-MCI group only showed more social contact activities (*t* = 5.01, *p* = 0.026). However, there were no significant differences in medical disease history and emotion between the NC-MCI and NC-NC groups.

**Table 4:** Characteristics of the study sample at baseline by incident MCI status.

**Characteristic**	**NC-MCI (*N* = 72)**		**NC-NC (*N* = 849)**		***t*-test/Chi-Square/*F*-test**	***p***
	**Mean**	**SD**	**Mean**	**SD**		
Age (year)	66.25	6.99	64.1	6.60	2.637	*p* = 0.009
Education (year)	10.88	3.43	11.66	3.12	−2.039	*p* = 0.042
Gender (M/F)	31/41		306/543		1.407	*p* = 0.236
BMI	24.84	3.65	24.54	3.15	0.516	*p* = 0.473
Married	63 (91.3%)		747 (89.1%)		0.312	*p* = 0.576
Smoking	53 (76.8%)		650 (79.0%)		0.179	*p* = 0.672
Drinking	32 (76.2%)		441 (77.5%)		0.039	*p* = 0.844
Jobscore	3.11	1.52	3.08	1.54	0.001	*p* = 0.974
Imcomelevel	5.58	2.61	5.41	2.44	0.244	*p* = 0.621
UCLA	35.06	8.49	34.58	9.13	0.377	*p* = 0.540
GDS	8.08	5.41	6.95	5.68	3.419	*p* = 0.065
Intellectual activities	1.95	0.55	2.15	0.61	1.780	*p* = 0.183
Physical activities	2.58	0.57	2.64	0.55	0.054	*p* = 0.816
Social contact activities	1.83	0.65	2.13	0.70	5.009	*p* = 0.026
Eating Habit	3.30	0.48	3.37	0.36	1.231	*p* = 0.270
Life regularity	3.61	0.45	3.56	0.39	0.200	*p* = 0.655
Diabetes mellitus	15 (21.7%)		132 (15.9%)		1.574	*P* = 0.210
Hypertension	34 (49.3%)		333 (40.2%)		2.186	*p* = 0.139
Hyperlipidemia	21 (42.9%)		263 (45.1%)		0.093	*p* = 0.761
Cerebrovascular disease	09 (13.0%)		76 (9.2%)		1.117	*p* = 0.291
Coronary heart disease	4 (5.8%)		95 (11.5%)		2.082	*p* = 0.149
Length of Follow up (y)	2.25	1.86	2.01	1.37	2.721	*p* = 0.099
MMSE	27.18	1.75	28.21	1.48	24.82	*p* < 0.001
Memory						
N5	4.53	2.13	6.34	2.17	35.96	*p* < 0.001
N1N5	25.88	7.25	32.70	7.92	38.75	*p* < 0.001
ROdelay	10.75	5.47	14.35	5.88	18.52	*p* < 0.001
Attention						
SDMT	31.30	9.13	37.05	10.60	9.03	*p* = 0.003
TMT-A	61.32	21.83	53.61	15.81	8.38	*p* = 0.004
Language						
VFT	41.54	8.41	46.83	8.03	21.40	*p* < 0.001
BNT	22.34	3.59	23.82	3.27	11.33	*p* = 0.001
Executive Function						
StroopC	83.96	23.32	73.86	23.24	8.16	*p* = 0.004
TMT-B	188.87	58.70	161.34	56.92	7.81	*p* = 0.005

At the baseline cognitive assessment, compared with the NC-NC group, the NC-MCI group showed poorer cognitive performance in memory (*F*_N5_ = 53.96, *p*_N5_ < 0.001; *F*_Rodelay_ = 18.52, *p*_Rodelay_ < 0.001), attention (*F*_SDMT_ = 9.03, *p*_SDMT_ = 0.003; *t*_TMTa_ = 8.38, *p*_TMTa_ = 0.004), language (*t*_VFT_ = 21.40, *p*_VFT_ < 0.001; *t*_BNT_ = 11.33, *p*_BNT_ = 0.001) and executive function (*t*_stroopCtime_ = 8.16, *p*_stroopCtime_ = 0.004; *t*_TMTb_ = 7.81, *p*_TMTb_ = 0.005; Table 4).

### Risk scores of incident MCI

Because the differences in baseline cognitive performance were significant between the two groups, we added cognitive performance to risk model 3 for predicting incident MCI. By comparing the RS1 and RS2 models, we found that RS2 with N1N5 (*B* = −0.073, *p* = 0.005), Rodelay (*B* = −0.072, *p* = 0.013), and VFT (*B* = −0.078, *p* = 0.001) scores had better performance, as shown in Supplementary Table S1. The AUC of the incident MCI model was 0.785, sensitivity = 0.717, and specificity = 0.830 (Figure 1B). According to model 3 and the coefficients, we can calculate the risk of incident MCI by the following formula:


$$P_{nc-mci}=\frac{e^{3.576-0.073\times N1N5-0.072\times Rodelay-0.0781\times VFT+0.084\times RS2}}{1+e^{3.576-0.073\times N1N5-0.072\times Rodelay-0.0781\times VFT+0.084\times RS2}}$$

When *p* > 0.1058, this person has a risk of incident MCI. The accuracy was 82.0%.

## Discussion

Our study found that (1): 1,174 subjects (19.8%) were diagnosed with MCI at baseline, and 72 (7.8%) of 849 developed MCI in the longitudinal studies; (2) the risk factors for MCI included age, education, cerebrovascular disease, and cognitive and physical activities; (3) the risk score, based on the above factors, had an area under the curve [receiver operating characteristic (ROC)] between 0.64 and 0.7; and (4) the final risk score model adding the cognitive assessment predicted MCI conversion more accurately (AUC = 0.785). Cognitive performance at baseline has predictive value regarding the conversion of MCI. Generally, the AUC values of model 2 and the final model were between 0.70 and 0.80, which can be regarded as good, especially when predicting incident MCI in the subsequent 3–5 years based on baseline information. The risk score values were derived from β coefficients of the logistic regression model, and our simple scoring approach did not result in important loss of information compared to original coefficients.

The risk score provides a quantitative estimation of the probability of conversion to MCI. Therefore, the score should be mainly used to target preventive measures in those most at risk, and it should not be used to label individuals as being MCI or non-MCI in the future. This scoring system is intended to be used as a practical tool. The calculation does not require extensive, specialized testing or expensive and/or labor-intensive procedures, such as a full neuropsychological test battery or brain imaging, to provide a prediction score (Exalto et al., 2013; Pankratz et al., 2015). A risk score is a screening tool to be used in the general population; therefore, predictors need to be noninvasive, inexpensive, and easily attainable. The other risk profile of AD in a large-sample cohort study included measures of age, marital status, BMI, stroke, diabetes, cardiovascular disease, and MRI white matter disease. The measures of the MCI risk score in our study also included age, education, sex, cerebrovascular disease, and intellectual and physical activities.

Age is the greatest risk factor for dementia and MCI. MCI usually presents in older age, with exponential increases in incidence after the age of 65 years (Suzuki et al., 2015). Age remains an important consideration, especially as life expectancy continues to increase. Higher education levels are associated with lower rates of MCI (Satizabal et al., 2016). Low educational level is thought to result in vulnerability to cognitive decline because it results in less cognitive reserve, which enables people to maintain function despite brain pathology (Valenzuela, 2008).

Our results are consistent with other observational reports that more frequent engagement in intellectual activity was related to a slower cognitive decline in old age (Wilson et al., 2007, 2013; Geda et al., 2011). Some epidemiological studies have found that a higher level of intellectual activity was associated with a lower risk of AD (Wilson et al., 2002, 2007) and incident MCI (Geda et al., 2011; Hussin et al., 2019). Based on the cognitive reserve hypothesis, which refers to the capacity of the brain to withstand the effects of pathological changes by recruiting alternative neurological processes or pathways (Valenzuela, 2008), engaging in more intellectual activity would have beneficial effects against cognitive decline (Valenzuela and Sachdev, 2006). However, a study using multiple neuroimaging modalities, including MRI and PET, took into account why there was better cognitive performance supported by lifelong intellectual activity and found that cognitive performance was independent from, rather than overlapping with, markers of neurodegeneration, including brain β-amyloid burden, brain glucose metabolism, or hippocampal volume (Gidicsin et al., 2015). Cognitive interventions in healthy older adults are associated with improvements in cognitive function (Ball et al., 2002; Willis et al., 2006).

Higher levels of physical activities (Beeri and Middleton, 2012; Buchman et al., 2012; Boripuntakul et al., 2014) are protective against dementia. The results of a meta-analysis of prospective cohort studies following individuals without dementia reported that physical activity had a significant protective effect against cognitive decline, with high levels of exercise being the most protective (Firth et al., 2018). Physical exercise leads to benefits in older people without dementia, such as improving balance and reducing falls (de Labra et al., 2015), reducing mortality and improving function and cognitive maintenance (Stephen et al., 2020).

In general, lifestyle activities such as intellectual and physical activities may lead to better cognitive performance due to more efficient cognitive networks and conditioning. More efficient cognitive networks could help older adult resist brain ageing and incident MCI or AD. Multidomain interventions have many cognitive benefits, which means that a healthy lifestyle may help reduce the risk of AD (Solomon et al., 2018).

Cognitive performance is the central component of an AD/MCI diagnosis. Thus, it is to be expected that cognitive performance is a sensitive predictor of conversion from MCI to AD (Belleville et al., 2017). A combination of measures from a range of domains typically provides a better predictor of disease progression. These neuropsychological measures had an overall accuracy of greater than 90% for the progression to AD (Tabatabaei-Jafari et al., 2018).

However, there are several limitations in this study. First, our analyses focused on MCI incidence without regard for MCI subtype. It is likely, for example, that risk factors may be different for aMCI and non-aMCI. We chose to focus this study on MCI more generally because of the potential clinical utility of these findings that are targeting general practitioners and lay people who do not have significant clinical training. Second, other potential risk factors for MCI, such as the APOE gene and amyloid β-protein in the brain, were not assessed in the examination. The inclusion of these additional factors, for which we did not have relevant information, may have further improved the predictive accuracy of the MCI risk score.

## Conclusion

Currently, there is no curative treatment for AD, which emphasizes the importance of primary prevention. The MCI risk score is a practical method for predicting the risk of MCI. This approach draws attention to the role of lifestyle factors in the development of MCI. Creating an easily understandable and practical scoring system that provides an estimated risk of MCI represents an important area of research. This risk estimation system not only allows general practitioners to utilize complex statistical models in a clinical setting but also helps individuals identify their potential risk profile and prevent or delay the future incidence of MCI and dementia. Research is still needed to validate and to further develop the MCI risk score in other populations.

## Data Availability Statement

The original contributions presented in the study are included in the article/Supplementary material, further inquiries can be directed to the corresponding author.

## Ethics Statement

The studies involving human participants were reviewed and approved by The ethics committee of the State Key Laboratory of Cognitive Neuroscience and Learning, Beijing Normal University. The patients/participants provided their written informed consent to participate in this study.

## Author Contributions

ZZ conceived the current study. XL, JZ, and KX performed the experiments. JX, YL, and XL analyzed the data. XL and KC wrote and revised the manuscript. All authors contributed to the article and approved the submitted version.

## Funding

This work was supported by Science and Technology Innovation 2030 Major Projects (grant number 2022ZD0211600), State Key Program of National Natural Science of China (grant number 82130118), Funds for International Cooperation and Exchange of the National Natural Science Foundation of China (grant number 81820108034), the Natural Science Foundation of China (grant number 32171085), The Fundamental Research Funds for the Central Public Welfare Research Institutes (grant number ZZ13-YQ-073), and The Fundamental Research Funds for the China Academy of Chinese Medical Sciences (grant number Z0601).
