# Supplementary material for: Risk scores of incident mild cognitive impairment in a Beijing community-based older cohort
Source: Front Aging Neurosci. 2022 Oct 3;14:976126. doi: 10.3389/fnagi.2022.976126 (PMC9574183; doi:10.3389/fnagi.2022.976126)
Supplement: Supplementary file 1 [file Table_1.docx]

**Supplementary Table S1** Cox models for MCI progression

|  | **B** | **S.E.** | **Wald** | **P** | **Exp(B)** |
| --- | --- | --- | --- | --- | --- |
| **N1N5** | -0.073 | 0.026 | 7.990 | 0.005 | 0.929 |
| **Rodelay** | -0.072 | 0.029 | 6.162 | 0.013 | 0.931 |
| **VFT** | -0.078 | 0.022 | 12.091 | 0.001 | 0.925 |
| **RS2** | 0.084 | 0.156 | 0.294 | 0.588 | 1.088 |
| **Intercept** | 3.576 | 1.299 | 7.576 | 0.006 | 35.736 |
